# Gene Expression Profiles of Multiple Synchronous Lesions in Lung Adenocarcinoma

**DOI:** 10.3390/cells10123484

**Published:** 2021-12-10

**Authors:** Jisun Lim, Yeon Bi Han, Soo Young Park, Soyeon Ahn, Hyojin Kim, Hyun Jung Kwon, Choon-Taek Lee, Sukki Cho, Jin-Haeng Chung

**Affiliations:** 1 Medical Research Collaborating Center, Seoul National University Bundang Hospital, Seongnam 13620, Korea; swanjslim@gmail.com; 2 Department of Pathology and Translational Medicine, Seoul National University Bundang Hospital, Seongnam 13620, Korea; 82580@snubh.org (Y.B.H.); stock75@snu.ac.kr (S.Y.P.); hyojinkim7137@snubh.org (H.K.); 89812@snubh.org (H.J.K.); 3 Department of Internal Medicine, Seoul National University Bundang Hospital, Seongnam 13620, Korea; ctlee@snu.ac.kr; 4 Department of Thoracic and Cardiovascular Surgery, Seoul National University Bundang Hospital, Seongnam 13620, Korea; tubincho@snu.ac.kr; 5 Department of Pathology, Seoul National University College of Medicine, Seoul 03080, Korea

**Keywords:** non-small cell lung cancer, lung adenocarcinoma, atypical adenomatous hyperplasia, RNA-seq, immune response

## Abstract

Many studies support a stepwise continuum of morphologic changes between atypical adenomatous hyperplasia (AAH) and lung adenocarcinoma (ADC). Here we characterized gene expression patterns and the association of differentially expressed genes and immune tumor microenvironment behaviors in AAH to ADC during ADC development. Tumor tissues from nine patients with ADC and synchronous multiple ground glass nodules/lesions (GGN/Ls) were analyzed using RNA sequencing. Using clustering, we identified genes differentially and sequentially expressed in AAH and ADC compared to normal tissues. Functional enrichment analysis using gene ontology terms was performed, and the fraction of immune cell types was estimated. We identified up-regulated genes (*ACSL5* and *SERINC2*) with a stepwise change of expression from AAH to ADC and validated those expressions by quantitative PCR and immunohistochemistry. The immune cell profiles revealed increased B cell activities and decreased natural killer cell activities in AAH and ADC. A stepwise change of differential expression during ADC development revealed potential effects on immune function in synchronous precursors and in tumor lesions in patients with lung cancer.

## 1. Introduction

Lung cancer is the leading cause of cancer-related deaths worldwide. The molecular basis and histology of lung cancer are heterogeneous [1]. Among patients presenting with non-small cell lung cancer (NSCLC), lung adenocarcinoma (ADC) is one of the most common subtypes [2]. In NSCLC, ADC is believed to follow a multistep progression [3,4,5]. Atypical adenomatous hyperplasia (AAH) is a precursor or a premalignant lesion of lung adenocarcinoma [6]. It is, therefore, imperative that a better understanding be achieved regarding the initiation of tumorigenesis in AAH to elucidate the mechanism underlying its progression to early invasive lung adenocarcinoma [7,8,9,10,11]. 

Recent technological advancements have improved our capacity to characterize the molecular profiles associated with disease states. For instance, high-throughput next generation genome sequencing has been exploited to define lung cancer molecular profiles. Moreover, differential gene expression analysis using RNA sequencing (RNA-seq) facilitates the identification of aberrant patterns in gene expression related to lung cancer development. Indeed, several studies have sought to characterize gene expression patterns and to associate these patterns with the immune response signatures of NSCLC [12,13,14]. One such study, using comprehensive transcriptional analysis, confirmed changes in T cell and natural killer (NK) cell composition during early lung adenocarcinoma [15]. However, although the gene expression profiles of ADC in the development of lung cancer have been well investigated [12,13], relatively little is known about the global transcriptome changes associated with AAH [6]. Moreover, although recent studies have reported suppressed antitumor and enhanced protumor activities related to immune signaling in AAH, as well as sequential changes in immunity-related features across a continuum from preinvasive to advanced lung ADC [10,16], these results require further verification. However, due to the scarcity of tissue samples and the highly diverse genetic background of lung ADC, confirmatory studies are challenging. 

In this study, we hypothesized that differentially expressed gene (DEG) profiles among adjacent histologically non-tumor (hereafter referred to as normal) lung tissue, AAH, and ADC tumors are associated with the molecular characterization of synchronous sequential lesions in peripheral pulmonary ADC and are related to the stepwise tumor progression observed in ADCs. Moreover, DEGs related to early lung adenocarcinoma have the potential to affect immune pathway activity. We, therefore, investigated the sequential changes in gene expression profiles of normal, AAH, and ADC samples and identified immune-related DEGs during the examination of surgical specimens harboring malignant tumors, using an integrated approach.

## 2. Materials and Methods

### 2.1. Patients and Specimen Procurement

Patient specimens were collected as part of a previous targeted sequencing study and the same patient identification numbers were used [8] (Table S1). Formalin-fixed, paraffin-embedded (FFPE) lung samples were obtained from patients who underwent wedge resection or a lobectomy for ADC at Seoul National University Bundang Hospital between January 2013 and December 2016 [8,17]. A total of nine patients with ADC and synchronous AAH were selected, and matched normal tissues were collected. Informed consent was obtained from all patients. This study was approved by the institutional review board of Seoul National University Bundang Hospital (IRB No. B-1607/355-301).

### 2.2. Sample Preparation

The selected FFPE lung samples were cut into six slices for ADC tissue and ten for AAH and normal tissue, each approximately 20 μm thick [8,17]. Board-certified pathologists reviewed hematoxylin and eosin-stained slides of each section to ensure that lesions were present in each specimen. Separated lesional regions were obtained by micro-dissection based on the pathologists’ reviews. Total RNA was extracted from FFPE tissues by deparaffinization, protease digestion, and TRIzol (Thermo Fisher Scientific, Waltham, MA, USA) extraction, according to the manufacturer’s instructions. 

To construct cDNA libraries with the TruSeq RNA Access Library Prep Kit (Illumina, Inc., San Diego, CA, USA), 1 µg of total RNA was used. The protocol included polyA-selected RNA extraction, RNA fragmentation, random-hexamer-primed reverse transcription, and 100 base pair paired-end sequencing with an Illumina HiSeq2500 sequencer (Macrogen, Inc., Seoul, Korea). The cDNA from the libraries was quantified using qPCR and qualitatively assessed using an Agilent Technologies 2100 Bioanalyzer (Agilent Technologies, Santa Clara, CA, USA). The RNA samples were aliquoted and stored at −80 °C for validation experiments. 

### 2.3. RNA-seq Processing

Sequencing reads were aligned to the human genome (hg19) using Bowtie 2 (v2.2.3). The quality of the reads was evaluated using FastQC (version 0.11.5) and trimmed with Trimmomatic (version 0.32). The number of raw reads for 27,684 known transcripts was quantified using HTseq (version 0.6.1p1) [18].

### 2.4. Statistical Analysis

Principal component analysis (PCA) of normalized data enabled visualization of sample and lesion heterogeneities. Read counts were normalized and analyzed to compare normal lung tissues to AAH tissues, normal lung tissues to ADC tissues, and AAH tissues to ADC tissues [19]. Cluster analysis of the DEGs was conducted using the STEM program (version 1.3.12) with 50 permutations per gene and a significance level of 0.05 [20]. DEGs were defined using three criteria: false-discovery rate (FDR) q-value ≤ 0.05, average fold change > 1.5 in at least one of the three pairwise comparisons, and STEM clustering profile *p*-value ≤ 0.05. We attempted to select genes that showed reliable expression levels across synchronous multiple lesions within a patient. To identify genes exhibiting significant monotonous trends across tissue types, DEGs were filtered using more stringent criteria (minimum 1.2-fold change for within-patient comparisons). The resulting DEGs were compared to those previously reported by Sivakumar et al. [10] for verification purposes.

Since the genomic variants from the same patient were available from the previous study, the expression patterns of *BRAF*-mutant and *KRAS*-mutant AAH samples were compared through the generation of a heatmap [8,17]. DEGs between *BRAF*- and *KRAS*-mutant AAH samples were extracted using the selection criteria of *p*-value < 0.01 and >1.5-fold change. 

Normalization and differential gene expression analyses were conducted with the DESeq2 package of the R program (version 3.5.3). Hierarchical clustering was performed based on a Pearson’s correlation coefficient algorithm and a complete method. Gene Ontology (GO) analysis and Kyoto Encyclopedia of Genes and Genomes (KEGG) pathway analysis were performed using DAVID (http://david.abcc.ncifcrf.gov, accessed on 17 April 2021).

### 2.5. Validation of RNA-seq Results with Quantitative PCR

We examined candidate genes (*ACSL5*, *SERINC2*, and *CCBE1*) that showed sequential gene expression patterns. First strand cDNA was generated with SuperScript II (Invitrogen) using random primers (Promega). Quantitative PCR (qPCR) primers were designed using Primer3 (http://www.ncbi.nlm.nih.gov/tools/primer-blast/, accessed on 17 April 2021), and qPCR was performed with the CFX Real-Time PCR System (Bio-Rad, Hercules, CA, USA) using Taq PCR mix (Roche, Basel, Switzerland), according to the manufacturer’s protocol. The PCR cycling conditions included an initial denaturation of 95 °C for 10 min, followed by 50 cycles at 95 °C for 10 s and 58 °C for 20 s, and 72 °C for 2 min. Comparative gene expression analysis was performed using the 2^−ΔΔCt^ method with data normalized to the level of human *GAPDH,* which was used as an internal control. These normalized values were represented as relative gene expression. The primer sequences used in this study for validation are presented in Table S2. 

### 2.6. Immunohistochemistry Analysis

To observe the localization and expression of the target proteins in lung tissue, immunohistochemistry (IHC) was performed using conventional heat-induced antigen retrieval procedures, as described previously [21]. Due to limited material, 32 samples were prepared. Protein expression and localization in lung tissue were analyzed using conventional heat-induced antigen retrieval procedures. The following primary antibodies were used: rabbit anti-human-ASCL5 (NBP2-31995, 1:50 dilution, Novus Biological, Centennial, CO, USA), rabbit anti-human-SERINC2 (NBP1-87927, 1:50 dilution, Novus Biological), rabbit anti-human-*DLX3* (LS-C176704, 1:50 dilution, LSBio, Seattle, WA, USA), rabbit anti-human-FUT2 (PA5-53159, 1:50 dilution, Thermo Fisher, Waltham, MA, USA), and rabbit anti-human-CCBE1 (PA5-59534, 1:50 dilution, Thermo Fisher). All slides were carefully reviewed by two of the authors (Y.B.H. and J.H.C.), and the stained area was considered positive if it included membranous staining alone or membranous and cytoplasmic staining together. 

### 2.7. Immune Signature Analysis and Immune-Related Transcriptome Profiles

To determine the compositional differences of specific immune cells in tumor lesions, we applied CIBERSORT (version 1.06) [22], a computational method that uses deconvolution methods to quantify the relative fractions of immune cell types from gene expression values [23]. We re-grouped 22 immune cell populations reported from CIBERSORT into 11 categories: B cells, plasma cells, CD8^+^ T cells, CD4^+^ T cells, T subsets (follicular helper T cells, regulatory T cells, and gamma delta T cells), NKs, monocytes and macrophages, dendritic cells, mast cells, eosinophils, and neutrophils. The cell population proportions between lesions were evaluated with a Student’s *t*-test. Furthermore, we assessed the gene expression profiles of the DEGs that overlapped with the 770 genes included in the nCounter PanCancer Immune Profiling Panel (NanoString Technologies, Seattle, WA, USA) to characterize their immune response features [24]. The overlapped genes were manually mapped to the immune-related functional categories and the GO biological processes provided by the PanCancer Panel manual, and the average fold change in each category was calculated. 

## 3. Results

### 3.1. RNA-seq Analysis of Tumor Samples from Lung Adenocarcinoma Patients

A total of 36 samples (18 AAH, 9 ADC, and 9 normal tissues) were available from 9 different patients. Within this patient cohort, there were three non-smokers, four ex-smokers, and two smokers. RNA-seq data analysis generated an average of 79,137,660 read counts with 95.12% mapping efficiency. Using PCA, we found that AAH samples were tightly clustered and located between normal lung tissue and ADC samples, while sample heterogeneity was partially explained by smoking (Figure S1, PCA analysis).

### 3.2. Identification of Differentially Expressed Genes

**Figure 1:**
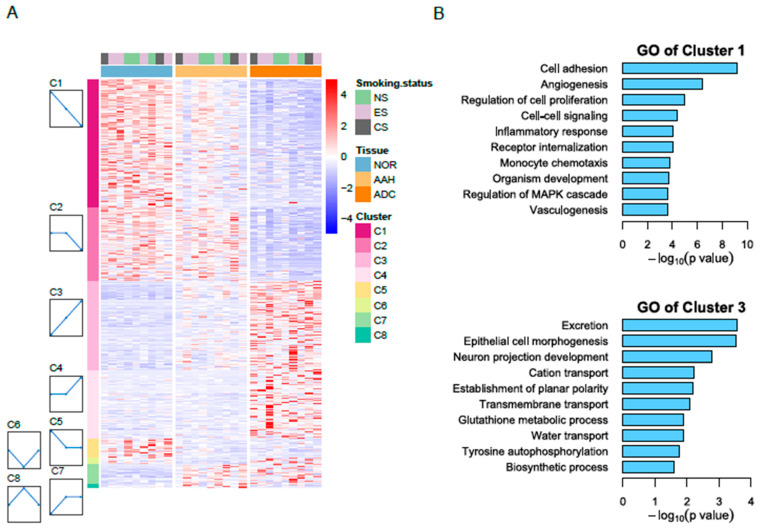
RNA-seq reveals differentially expressed genes in three tissue types. (**A**) Heatmap of differentially expressed genes in normal, AAH, and ADC tissues. Differentially expressed genes (DEGs) were grouped into eight clusters based on distinct gene expression profiles, namely C1 to C8. (**B**) Gene Ontology (GO) biological process results for Cluster 1 with decreasing pattern from normal to ADC (**top**) and for Cluster 3 with increasing pattern (**bottom**). AAH, atypical adenomatous hyperplasia; ADC, adenocarcinoma; CS, current smokers; ES, ex-smokers; NS, non-smokers.

We conducted all pairwise comparisons of normal lung tissue, AAH, and ADC, leading to the identification of 2741 DEGs in the pairwise comparisons (Figure S2, which shows a workflow for gene expression analysis). To further characterize sequential changes from normal lung tissue to AAH and ADC, we classified the expression profiles using the cluster method, which grouped DEGs into eight clusters based on distinct gene expression profiles (Figure 1A). Clusters 1–4 were most highly ranked according to the *p*-value of the permutation test using STEM. Cluster 1 mapped 863 genes (31%) that had a gradually decreased expression pattern. Cluster 2 included 497 genes (18%) that did not show a significant expression change between normal tissue and AAH; however, the genes in this cluster did exhibit decreased expression from AAH to ADC. Cluster 3 included 594 genes (22%) with a gradually increased expression pattern, and Cluster 4 included 463 genes (17%) with minimal expression changes from normal to AAH and subsequent increased expression from AAH to ADC. GO analysis further revealed that the genes in Cluster 1 were enriched in cell adhesion, angiogenesis, the regulation of cell proliferation, cell–cell signaling, and the inflammatory response (Figure 1B). Cluster 2 was primarily enriched in the cell surface receptor signaling pathway, angiogenesis, and the cellular response to tumor necrosis factor. Cluster 3 was enriched in genes associated with excretion, epithelial cell morphogenesis, neuron projection development, transmembrane transport, and tyrosine autophosphorylation. Cluster 4 was enriched in mitotic nuclear division, cell division, and sister chromatid cohesion.

### 3.3. Candidate Genes with Linear Changes across Tumor Lesions and Validation of Candidate Genes by qPCR and Immunohistochemistry

**Figure 2:**
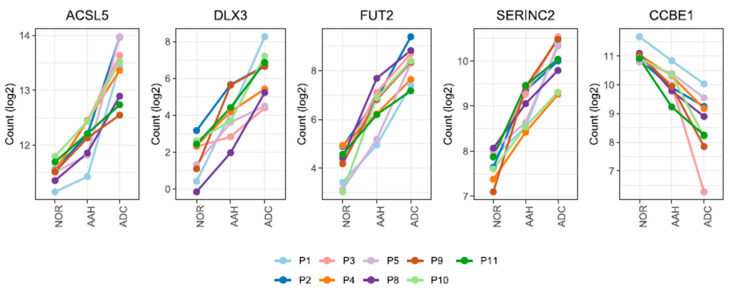
The mRNA expression patterns of five genes from normal and lung cancer tissue samples. Five genes are differentially expressed with a 1.2-fold change (AAH to normal tissue, ADC to normal tissue, and ADC to AAH) in all nine patients. Only FUT2 remained as the candidate gene when a fold-change cut-off of 1.5 was applied. The patient specimens and the numbers were obtained from a previous study [8,17]. Read count values (log2) are presented for 36 samples across 3 different tissues. AAH, atypical adenomatous hyperplasia; ADC, adenocarcinoma; NOR, normal.

We identified five candidate genes that satisfied the stringent criteria for 1.2-fold change in all pairwise comparisons and across all patients (Figure S2, which shows a workflow for gene expression analysis). The expression of *ACSL5*, *DLX3*, *FUT2*, and *SERINC2* gradually increased, while that of *CCBE1* gradually decreased during lesion development (Figure 2 and Figure S2). 

*DLX3* and *FUT2* showed low expression in normal tissue and remarkably high expression in ADC (5-fold and 4-fold changes in ADC compared to normal tissue, respectively). *ACSL5* and *SERINC2,* however, showed moderately enhanced expression in ADC compared with normal tissue (2-fold change and 2-fold change, respectively). *CCBE1* was down-regulated (2-fold change; Figure 2 and Table S2 present DEG fold changes calculated by DESeq2). The expression of three genes (*ASCL5*, *SERINC2,* and *CCBE1)* was validated by qPCR; the sequential changes in expression patterns were similar to our findings (Figure S3, which presents qPCR results). 

To confirm protein expression and localization in lung tissue, immunohistochemistry was performed for five selected genes (Figures S4–S8, which present microscopy images of specimens using IHC; Table S3, which shows H-scores). ACSL5, FUT2, and SERINC2 showed a gradual increase in expression across different tissue types. ACSL5 expression was observed in normal type II pneumocytes with cytoplasmic staining (Figure S4). In AAH, slightly more intense cytoplasmic staining was observed, with the strongest cytoplasmic and membranous staining observed in ADC samples. FUT2 expression was observed in normal bronchial epithelium and alveolar macrophages with weak cytoplasmic staining; however, no FUT2 expression was detected in normal type II pneumocytes (Figure S5). In AAH, pneumocytes exhibited cytoplasmic staining with moderate intensity, and in ADC, stronger staining intensity was observed. Moderate cytoplasmic SERINC2 expression was observed in normal bronchial epithelium, type II pneumocytes, and alveolar macrophages (Figure S6). Similarly, SERINC2 expression intensity also gradually increased in lesions that represented the transition from AAH to ADC. In ADC, diffuse and strong SERINC2 expression patterns were observed.

DXL3 labeling was performed in two cases (Figure S7). One case (P2) showed an increasing expression pattern similar to the pattern observed in the RNA-seq analysis, while in the other case (P4), AAH exhibited a stronger DXL3 expression pattern than ADC. CCBE1 expression was observed only in the respiratory epithelium, not in type II pneumocytes (Figure S8). Therefore, changes in CCBE1 expression could not be observed.

We performed an over-representation analysis using ConsensusPathDB (CPDB) on the five genes, and metabolism was reported as a statistically significant pathway (q = 0.003; source, Reactome; member in the metabolism pathway: *ACSL5*, *FUT2*, and *SERINC2*).

### 3.4. Gene Expression Profiles of BRAF and KRAS Genomic Mutations in AAH

We identified three AAH samples harboring the *BRAF* mutation (one in P3 and two in P8) and four AAH samples with the *KRAS* mutation (one in P2, two in P8, and one in P11) [8]. After exploring expression patterns, the average gene expression value was calculated from two AAH P8 samples harboring the *KRAS* mutation. Hierarchical clustering showed distinct genetic expression differences between *BRAF*-mutant and *KRAS*-mutant AAHs (Figure S9 presents a gene expression heatmap). 

We compared the gene expression clusters from a previously published report [10] with the clusters derived in our study and identified 269 genes (46% of 585 common genes between studies) with the same expression patterns (Table S4). The overlapping genes (*LRRC32*, *SEMA4A*, *ADAM23*, *NR1D2*, and *SCUBE2*) were identified. *LRRC32* and *SEMA4A* were highly expressed in *BRAF*-mutant AAH, whereas *ADAM23*, *NR1D2*, and *SCUBE2* were highly expressed in *KRAS*-mutant AAH (Figure S9). DEGs found to be associated with the small cell lung cancer pathway (*ITGA2*) and other cancer pathways (*MMP1*), based on KEGG analysis, were highly expressed in *KRAS*-mutant AAH (Figure S9) [25]. Overall, our findings were concordant with those of the earlier study [10]. 

### 3.5. Estimated Immune Response in Multiple Lesions

**Figure 3:**
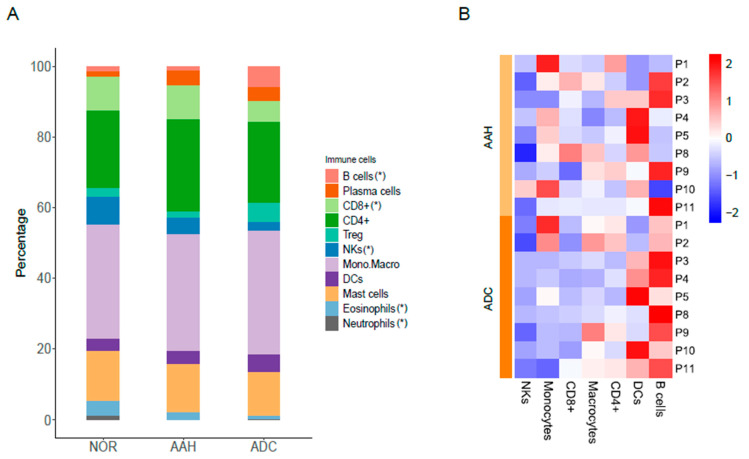
Immune cell signatures across different tissues and patients. (**A**) Proportion of immune cells in normal tissues, AAH, and ADC tissues. (*) indicates the significance test results (*p*-value < 0.05) in paired *t*-test of B cells (ADC vs. NOR), CD8+ (ADC vs. NOR), NKs (AAH vs. NOR, ADC vs. NOR, ADC vs. AAH), eosinophils (AAH vs. NOR, ADC vs. NOR, ADC vs. AAH) and neutrophils (AAH vs. NOR, ADC vs. NOR). (**B**) Relative differences expressed in z-score in tumor lesions compared to normal samples. AAH, atypical adenomatous hyperplasia; ADC, adenocarcinoma; NOR, normal.

We identified compositional differences in specific immune cells within multiple synchronous lesions in lung adenocarcinoma. The composition of immune cells (e.g., B cells, plasma cells, CD8^+^ T cells, CD4^+^ T cells, T subsets, NKs, monocytes and macrocytes, dendritic cells, mast cells, eosinophils, and neutrophils) across tissues and patients is indicated in Figure 3. CD8^+^ T cells were abundant in normal tissues and significantly reduced in ADC (8.99% to 5.79%, *p* = 0.001), which is in accordance with previous reports [13,15,26]. NKs were abundant in normal tissues and consistently reduced in AAH (8.66% to 4.52%, *p* = 0.027) and ADC (8.66% to 2.15%, *p* = 0.002). In contrast, B cells, which accounted for a low proportion in normal tissues, gradually increased in AAH and ADC (1.12% to 5.57%, *p* = 0.019), which is consistent with previous reports [15,22]. The neutrophil proportion decreased in ADC (1.15% to 0.4%, *p* = 0.09) and was found to be lower than that of B cells, which is consistent with results of a previous report [22]. The proportion of macrophages in normal tissue was not significantly altered in AAH (*p* = 0.96) and ADC (*p* = 0.24). 

**Figure 4:**
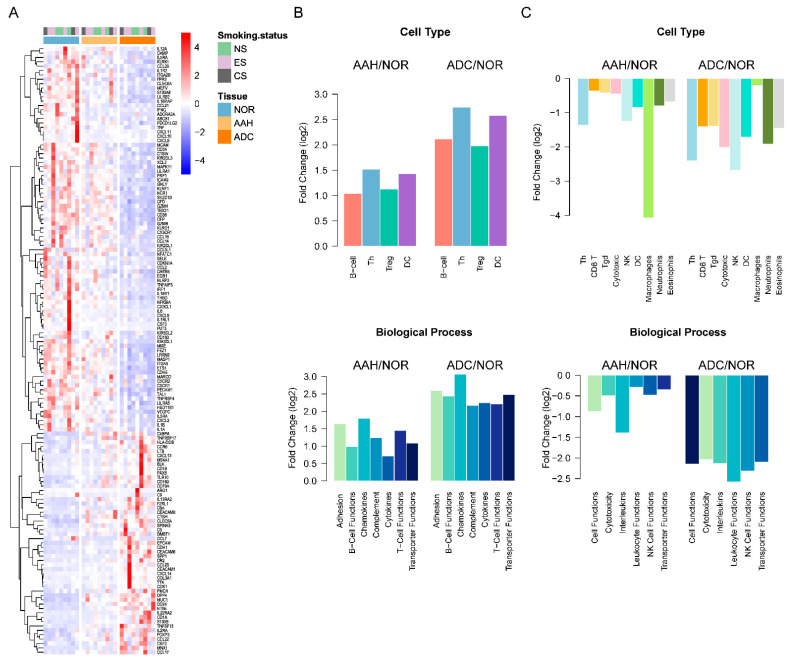
Immune signatures of differentially expressed genes (DEGs) in normal, AAH, and ADC tissues. (**A**) Heatmap of DEGs common in the PanCancer Panel. Samples are displayed in columns, while mRNAs are presented in rows. AAH, atypical adenomatous hyperplasia; ADC, adenocarcinoma; CS, current smokers; ES, ex-smokers; NOR, normal tissues; NS, non-smokers. (**B**) The average fold change of up-regulated DEGs in AAH and ADC compared to normal tissues grouped by immune cell types and Gene Ontology (GO) biological processes. (**C**) The average fold change in down-regulated DEGs in AAH and ADC tissues compared to normal tissue, grouped by immune cell types and GO biological processes.

To further investigate immune-related DEG expression profiles, we investigated 145 overlapping DEGs with the PanCancer Panel (Table S5). Among the 139 genes determined to be associated with immune-related functions, 48 were up-regulated in AAH or ADC compared to normal tissues (Table S6). The expression of these up-regulated genes was significantly increased in ADC compared to AAH or normal tissue samples, as shown in Figure 4A. Moreover, after mapping the up-regulated genes to immune cell categories and GO biological processes, we found that they were enriched in adhesion, B cell functions, chemokines, complement, cytokines, T cell functions, and transporter function, with an evident incremental pattern, as shown in Figure 4B (Table S6). For B cell functions, four genes (*BLK*, *CD19*, *CR2*, and *MS4A1*) were expressed in Cluster 3, where the gene expressions of AAH and ADC were higher than that of normal tissues. Similarly, we identified 91 down-regulated DEGs, which were found to be enriched in leukocyte functions, NK cell functions, and cytotoxicity, as shown in Figure 4C (Table S7). NK cell functions are enriched with the genes *IL12A*, *KIR2DL1*, *KIR2DL3*, *KIR3DL1*, *KIR3DL2*, *KLRD1*, *KLRF1*, *KLRK1*, and *NCR1*. *IL12A*, *KIR3DL2*, and *KLRK1* were expressed in Cluster 1 and *KIR2DL1*, *KIR2DL3*, *KIR3DL1*, *KLRD1*, *KLRF1*, and *NCR1* were expressed in Cluster 2. The genes in Cluster 1 had lower expressions in AAH and ADC than in normal tissues.

## 4. Discussion

The objective of this study was to conduct a comprehensive analysis of the gene expression profiles in preinvasive AAH and invasive ADC lesions that are associated with early lung adenocarcinoma development and to characterize the changes in their immune responses. To this end, we identified candidate genes with specifically increased or decreased expression patterns across normal, AAH, and ADC tissue samples, while validating the observations for *ACSL5* and *SERINC2* in independent qPCR experiments and IHC analysis. *FUT2* was enriched using qPCR.

Acyl-CoA synthetase long-chain (ACSL) family members are enzymes that catalyze the activation of 12–22 carbon long-chain fatty acids. Abnormal lipid synthesis and extracellular lipid uptake are advantageous modifications for cancer cell proliferation [27], and ACSLs play key roles in uncontrolled cancer cell proliferation. Accordingly, *ACSL5* is up-regulated in several cancers, including colorectal, breast, bladder, esophageal, lung, pancreatic, prostate, and malignant gliomas [27,28]. Moreover, the observed gradual increase in *ACSL5* expression as tissue changes from normal to AAH and ADC may support the notion that *ACSL5* contributes to cancer progression. However, Chen et al. [28] reported that patients with breast, colorectal, lung, and ovarian cancer with higher *ACSL5* expression showed good survival outcomes. Therefore, the association between *ACSL* expression and malignancy requires more in-depth analysis.

*FUT*s play a role in important glycosylation events in cancer [29]. Up-regulation of *FUT2* has been observed in non-small cell lung cancer, lung adenocarcinoma, and small cell lung cancer [29,30,31].

The SERINC family of transmembrane proteins incorporates serine into membrane lipids during membrane synthesis. *SERINC2* overexpression has been reported to be significantly associated with AAH and ADC [32,33,34,35,36]. Furthermore, Zeng et al. reported that *SERINC2*-knockdown suppresses lung adenocarcinoma proliferation, migration, and invasion through a mechanism that may be associated with phosphatidylinositol 3-kinase/AKT signaling [36]. Based on these findings, *SERINC2* might be required for lung adenocarcinoma progression.

We showed that the metabolism pathway was shared by *ACSL5*, *FUT2*, and *SERINC2*. A recent study [37] has reported that the metabolic pathways emerge in premalignant lesions and that metabolite-based clustering showed different survival outcomes, possibly facilitating early detection of high-risk cancers. Our results revealed the candidate genes linked to the evidence that metabolic pathways emerge in premalignant lesions. Although the expression levels of *FUT2* and *SERINC2* were not related to prognosis (data not shown), their over-expression pattern in tumors is reproducible. Collectively, our findings facilitate the identification of diagnostic markers for lung cancer.

The results of several studies investigating the impact of immunotherapeutic treatment on patients with advanced-staged NSCLC have demonstrated significant clinical improvement [38,39]. However, surgical intervention is a standard treatment for early-stage NSCLC, while the introduction of immunotherapy into the treatment of early-stage NSCLC remains in the experimental stages [40]. Multiple clinical trials are underway to explore the effects of neoadjuvant or adjuvant treatment with ICIs. Although surgery is a standard treatment for early-stage NSCLC and results from a large-scale phase III trial are not yet available, the data from some phase I–II trial results appear promising (PMID [34234606][34685407]). For example, the Lung Cancer Mutation Consortium (LCMC3, NCT02927301) study, a phase II trial investigating the safety of atezolizumab as a neoadjuvant and adjuvant treatment in 181 patients with stage IB–IIIB resectable NSCLC, achieved the primary study objective of a major pathologic response of 21% and a pathologic complete response of 7%, and no substantial safety issue has been reported [41]. Other trials (Checkmate 159 [NCT02259621], NEOSTAR [NCT03158129], and MK3475-223 [NCT02938624]) have similarly showed favorable safety and efficacy results pertaining to neoadjuvant ICI treatment in early-stage NSCLC. Our findings suggest that complex compositional changes occur in immune cells during the initiation of preinvasive lesion development. Specifically, compared to normal tissues, B cell signature activity increased while CD8^+^ T cell and NK cell activities decreased in AAH and ADC. Furthermore, with the DEGs that overlapped with the PanCancer Immune Panel, the enriched GO categories indicated that the up-regulated DEGs were enriched in chemokines, B cell functions, and cytokine signaling in the immune system, and the down-regulated DEGs were enriched in interleukins, leukocyte functions, and NK cell functions. The overexpression of the genes in B cell functions in lung cancer has been reported [42,43,44]. Increased expression levels of BLK [42] and MS4A1 [43] have been reported in lung adenocarcinoma, and BLK and CD19 [44] have been reported to be significantly correlated with the overall survival in patients with lung adenocarcinoma. Some studies have reported decreased expressions of the genes in NK cell functions in lung cancer. The expression levels of IL12A [10] and KIR3DL2 [45] in lung adenocarcinoma, and KIR3DL2 in lung squamous cell carcinoma were significantly lower than in adjacent normal tissues and decreased as the clinical stage increased. KLRK1 expression in NSCLC was significantly lower than in normal tissues, indicating a prognostic gene [46].

There are two major limitations in this study. First, a small number of cases may restrict generalization of the results, although we employed paired-tissue sampling to minimize the between-patient variability. Second, the study focused on monotonous changes over normal to AAH, and to ADC with a stringent cut-off; therefore, our candidate gene lists may not represent the full spectrum of genes playing a functional role in invasive malignancy.

In summary, this study presents a comprehensive analysis of the gene [43] expression profiles for lung adenocarcinoma with synchronous AAH. We also validated sequentially up-regulated genes (*ACSL5* and *SERINC2*) across AAH to ADC and identified differential immune cell profiles in AAH. These results support the hypothesis that cancer cell proliferation-related gene expression levels change, while the immune microenvironment becomes altered during the early phase of lung adenocarcinoma development.

## Data Availability

The datasets analyzed during the current study are available from the corresponding author on reasonable request.

## Supplementary Materials

The following are available online at https://www.mdpi.com/article/10.3390/cells10123484/s1, Table S1: Patient characteristics and specimen information, Table S2: Primer sequences, Table S3: Histochemical scoring assessment (H-score), Table S4: Differential expression of genes that overlapped with the 2017 study results of Sivakumar et al. (*n* = 269), Table S5: Differentially expressed genes that overlapped with the PanCancer Immune Profiling Panel gene lists (*n* = 145), Table S6: Annotated biological process of up-regulated DEGs that overlapped with the PanCancer Panel (*n* = 48), Table S7: Annotated biological process of down-regulated DEGs that overlapped with the PanCancer Panel (*n* = 91), Figure S1: Principal component analysis (PCA) plot of RNA-seq samples, Figure S2: Workflow of identification of candidate genes for mRNA expression analysis, Figure S3: Differential gene expression across three tissue types and nine patients, Figure S4: ASCL5 protein expression is elevated in AAH and ADC, Figure S5: FUT2 protein expression in normal, AAH, and ADC tissues, Figure S6: SERINC2 protein expression in adjacent non-tumor tissues and the associated AAH and ADC tissues, Figure S7: DXL3 protein expression visualized with immunohistochemistry, Figure S8: CCBE1 protein expression visualized using immunohistochemistry in P18 (normal and ADC), Figure S9: Differential gene expression in AAH samples with *BRAF* or *KRAS* mutations.
