# Supplementary material for: Gene Expression Profiles of Multiple Synchronous Lesions in Lung Adenocarcinoma
Source: Cells. 2021 Dec 10;10(12):3484. doi: 10.3390/cells10123484 (PMC8700398; doi:10.3390/cells10123484)
Supplement: Supplementary file 1 [file cells-10-03484-s001.zip › cells-supplementary figures_20211130.pdf]

Supplementary Figure S1

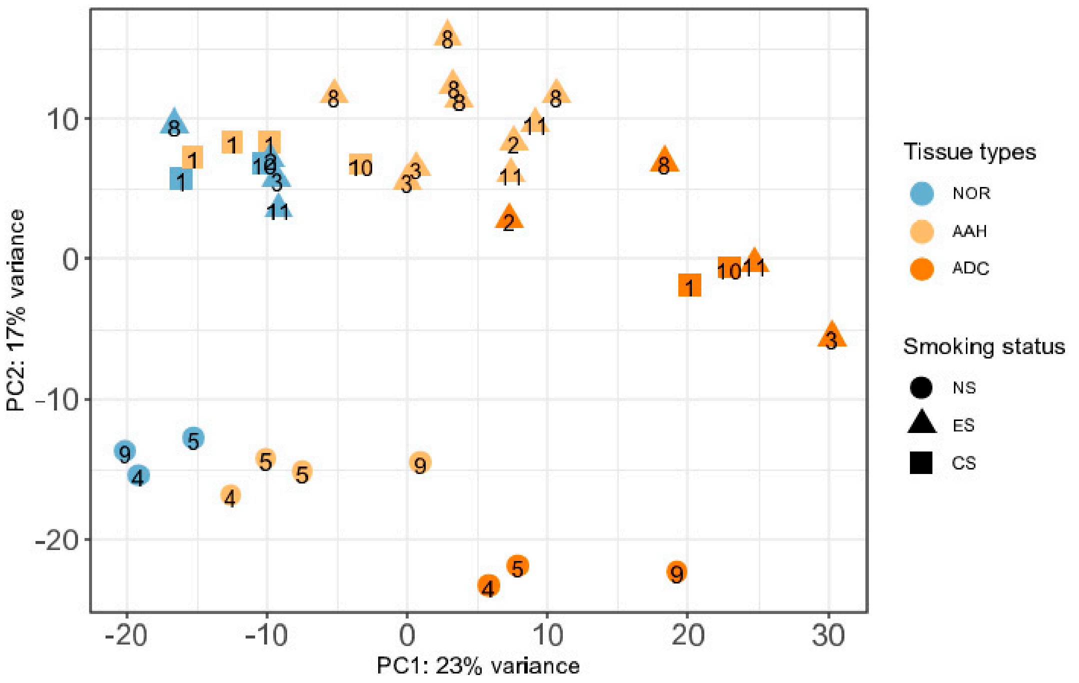

Figure S1. Principal component analysis (PCA) plot of RNA-seq samples. AAH, atypical adenomatous hyperplasia; ADC, adenocarcinoma; NOR, normal. CS, current smokers; ES, ex-smokers; NS, non-smokers. Numbers represent sample IDs used in Park *et al.* (2018).

Supplementary Figure S2

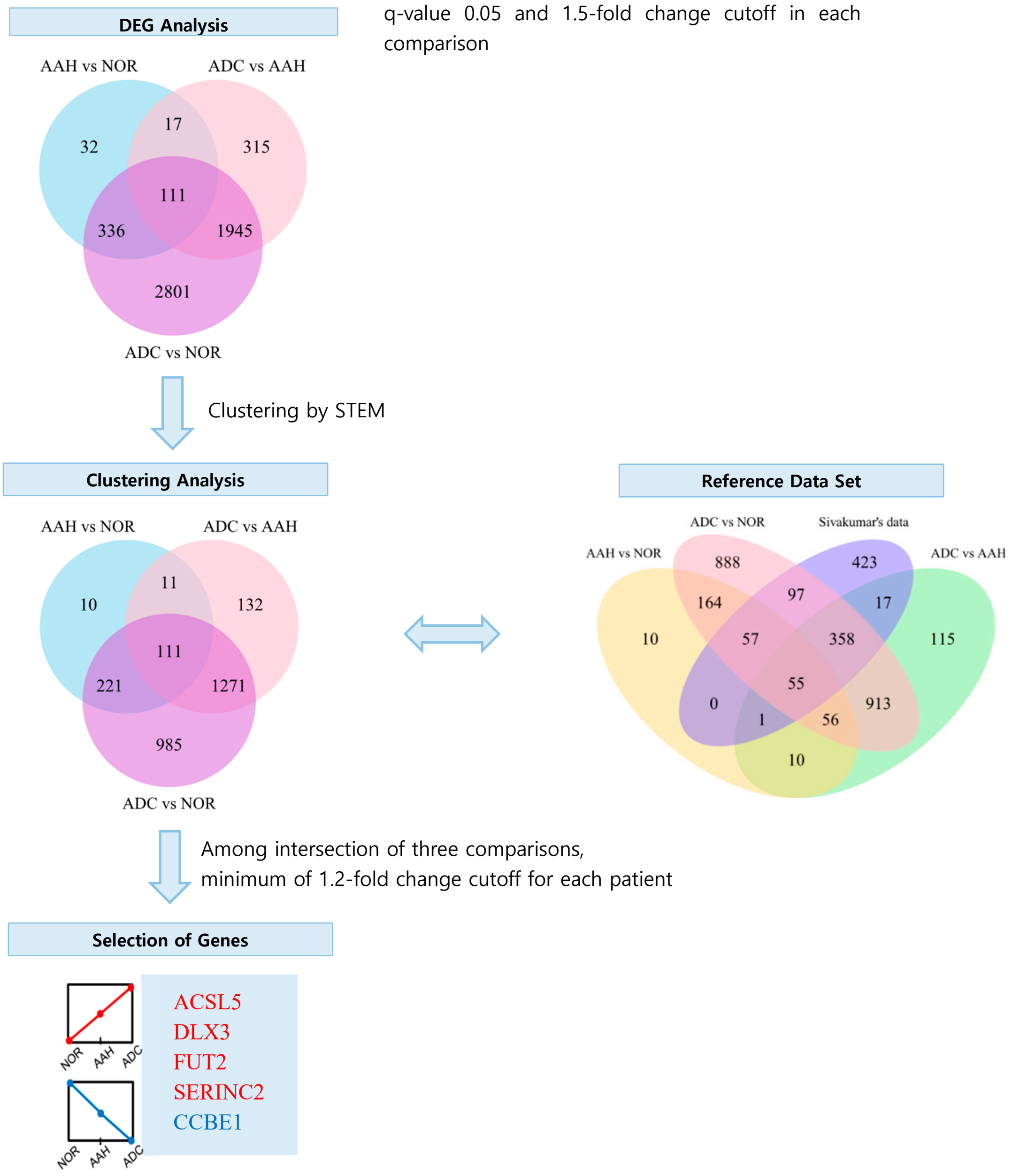

Figure S2. Workflow of identification of candidate genes for mRNA expression analysis Reference data set is collected from DEGs in Sivakumar *et al.*, 2017's data set. AAH, atypical adenomatous hyperplasia; ADC, adenocarcinoma; NOR, normal; FC, fold-change.

Supplementary Figure S3

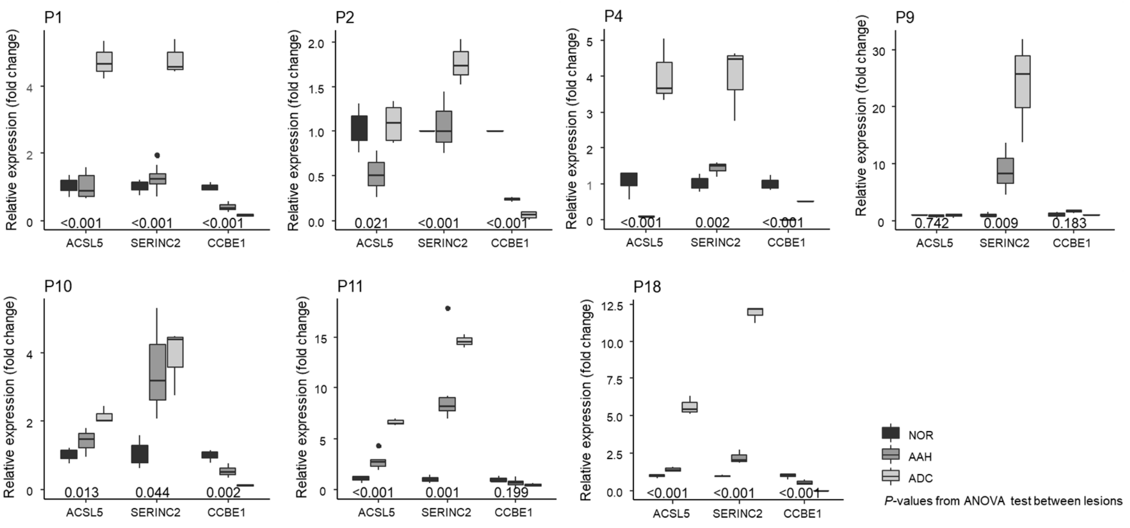

Figure S3. Differential gene expression across three tissue types and nine patients. Gene expression profiles were experimentally validated using qPCR. AAH, atypical adenomatous hyperplasia; ADC, adenocarcinoma; NOR, normal.

Supplementary Figure S4

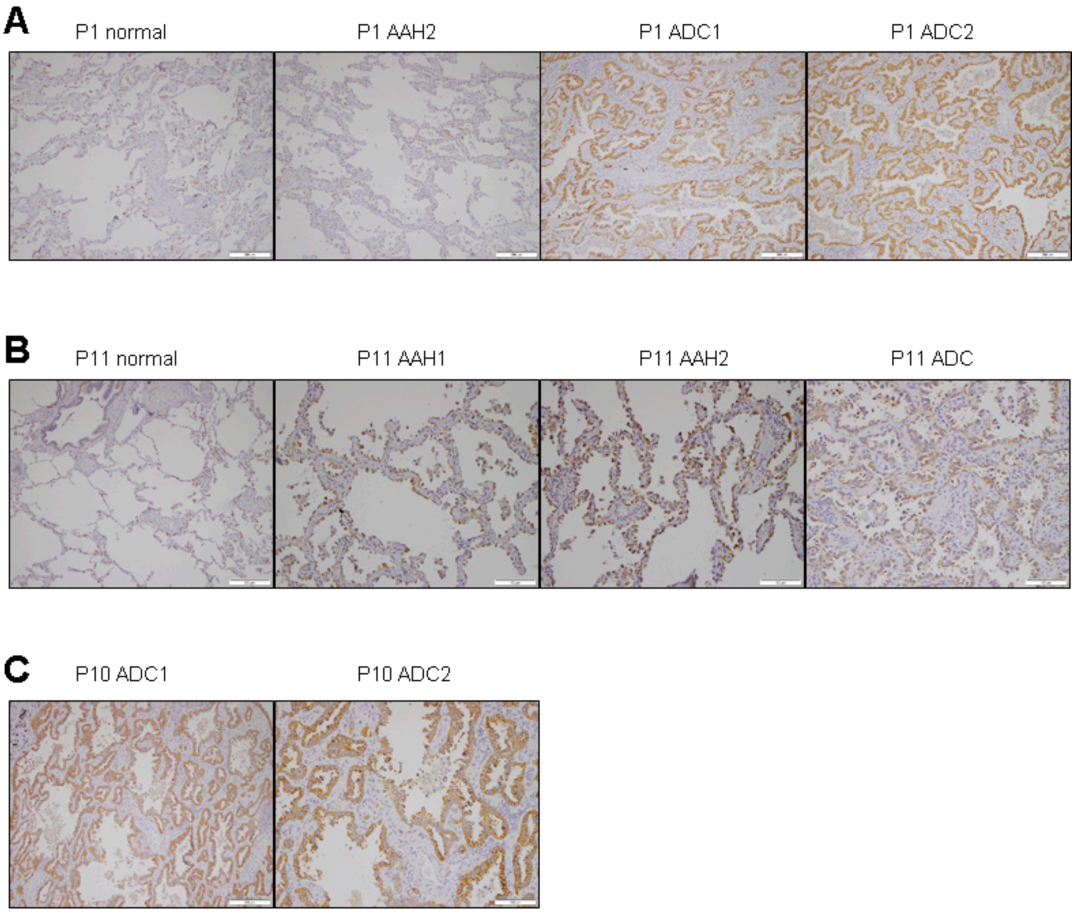

Figure S4. ASCL5 protein expression is elevated in AAH and ADC. (A) P1, AAH has partial staining patterns similar to normal tissues. ADC shows strong diffuse patterns (normal [×100], AAH [×100], ADC1 [×100], and ADC2 [×100]). (B) P11 has an overall increased labeling pattern compared to normal tissue samples and AAH, to ADC. (normal [×100], AAH1 [×200], AAH2 [×200], and ADC [×200]). (C) P10, ADC shows diffuse staining with a membranous pattern and strong intensity. (ADC1 [×100], and ADC2 [×200]). AAH, atypical adenomatous hyperplasia; ADC, adenocarcinoma; NOR, normal.

Supplementary Figure S5

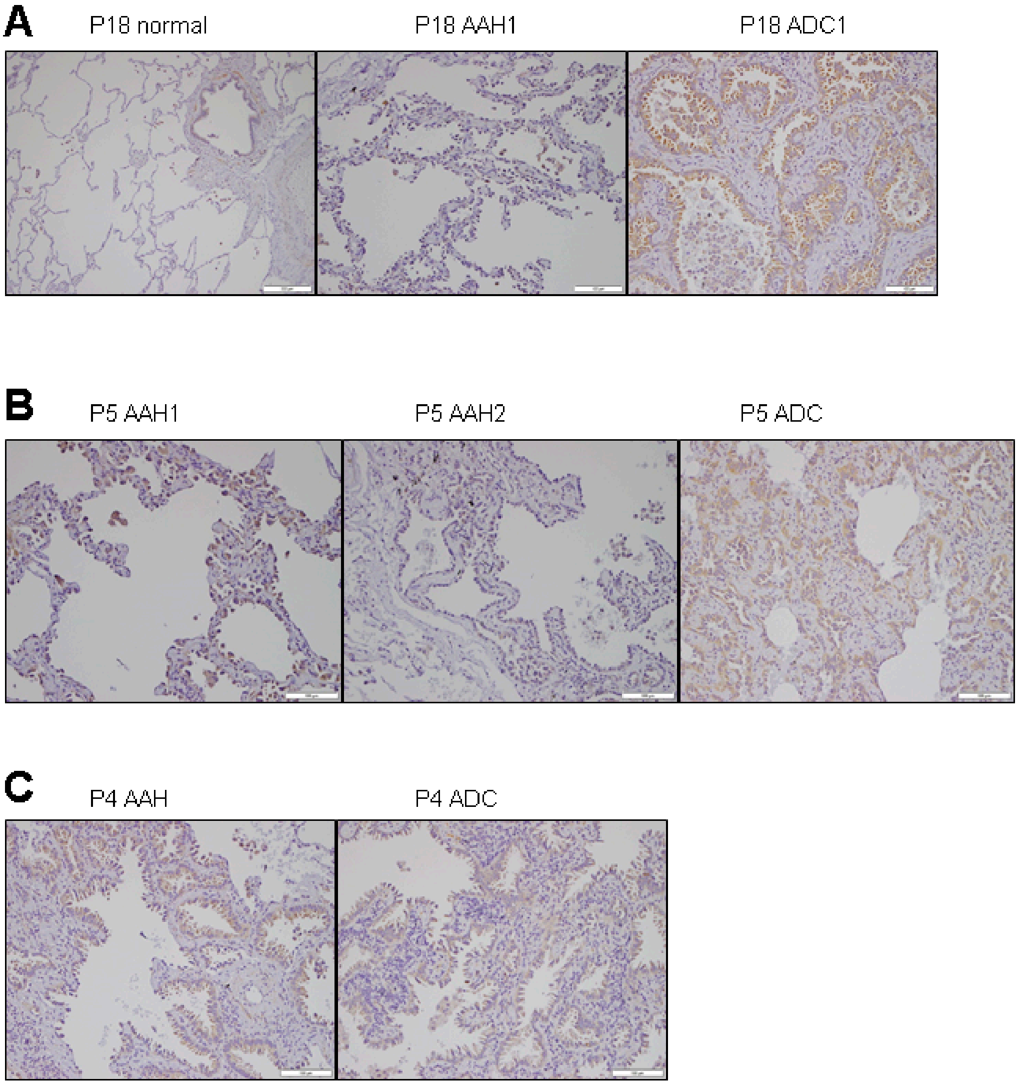

Figure S5. FUT2 protein expression in normal, AAH, and ADC tissues. (A) P18: AAH tissue has less FUT2 in pneumocyte cytoplasm, while ADC has consistently higher protein expression (normal [×100], AAH [×200], and ADC [×200]). (B) P5: AAH tissue has less staining in the pneumocyte cytoplasm, while ADC has consistently higher FUT2 expression (AAH1 [×200], AAH2 [×200], and ADC [×200]). (C) P4: AAH and ADC tissues have similar FUT2 protein expression patterns (AAH [×200], and ADC [×200]). AAH, atypical adenomatous hyperplasia; ADC, adenocarcinoma; NOR, normal.

Supplementary Figure S6

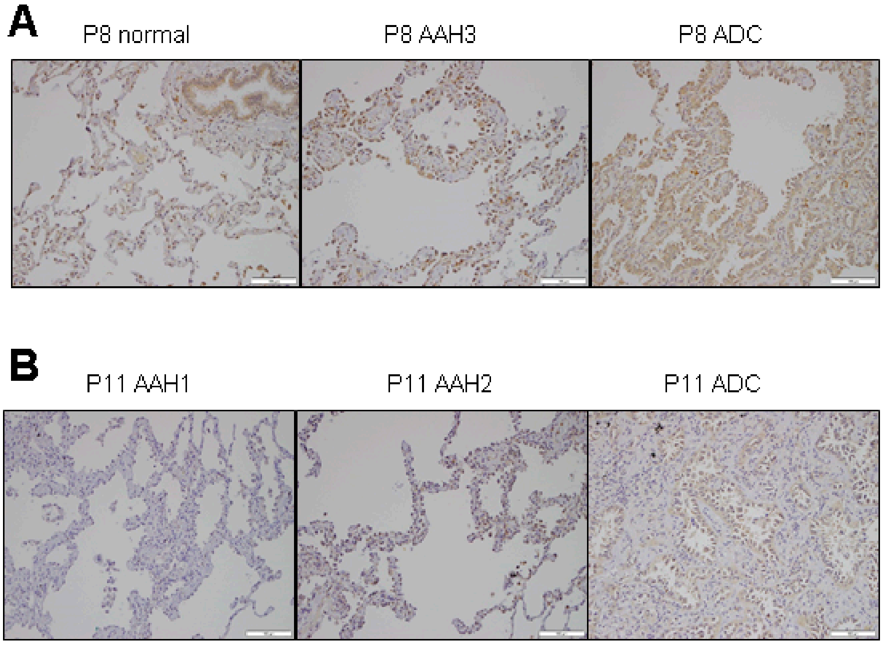

Figure S6. SERINC2 protein expression in adjacent non-tumor tissues and the associated AAH and ADC tissues. (A) P8: SERINC2 expression increases in intensity from normal respiratory epithelium, to AAH and ADC (normal [×200], AAH [×200], and ADC [×200]). (B) P11: ADC tissues show a diffuse SERINC2 expression pattern that is stronger than in AAH (AAH1 [×200], AAH2 [×200], and ADC [×200]). AAH, atypical adenomatous hyperplasia; ADC, adenocarcinoma; NOR, normal.

Supplementary Figure S7

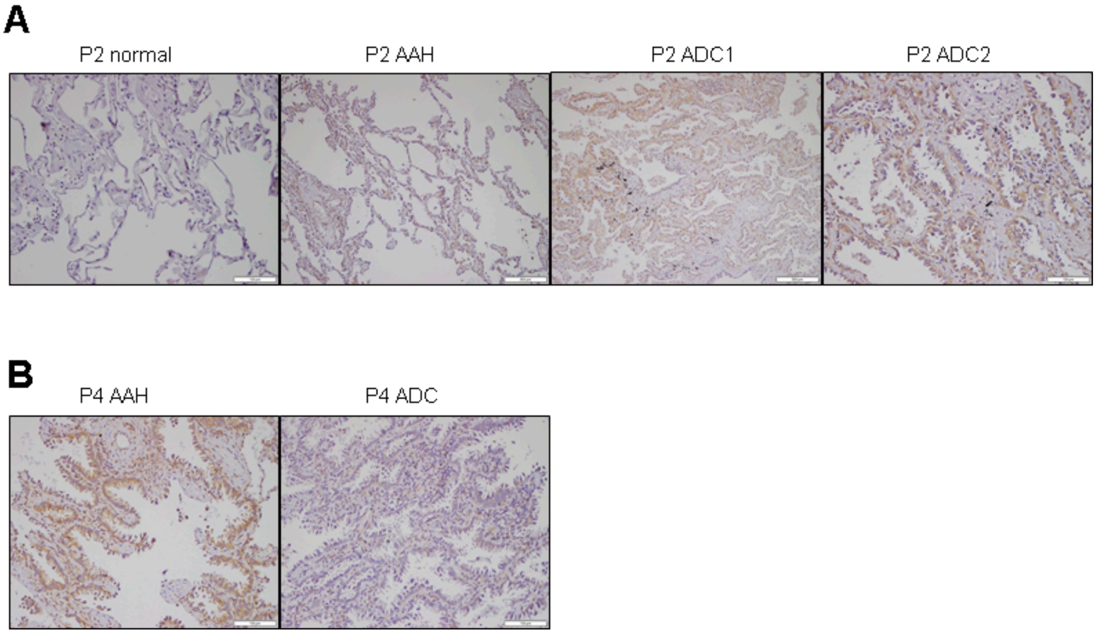

Figure S7. DXL3 protein expression visualized with immunohistochemistry. (A) P2: cytoplasmic staining is apparent and is increased in intensity in AAH and ADC samples compared to the control (normal [×200], AAH [×100], ADC1 [×100], and ADC2 [×200]). (B) P4: cytoplasmic staining is apparent in both tissue samples; however, it is higher in the AAH samples (AAH [×200], and ADC [×200]). AAH, atypical adenomatous hyperplasia; ADC, adenocarcinoma; NOR, normal.

Supplementary Figure S8

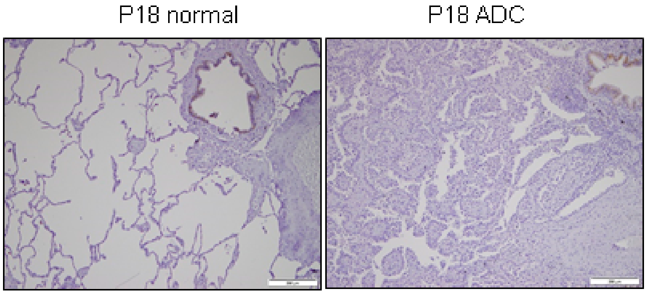

Figure S8. CCBE1 protein expression visualized using immunohistochemistry in P18 (normal, and ADC). There was a lack of staining in ADC, and the relative intensity between normal and ADC samples is not confirmed. ADC, adenocarcinoma; NOR, normal.

Supplementary Figure S9

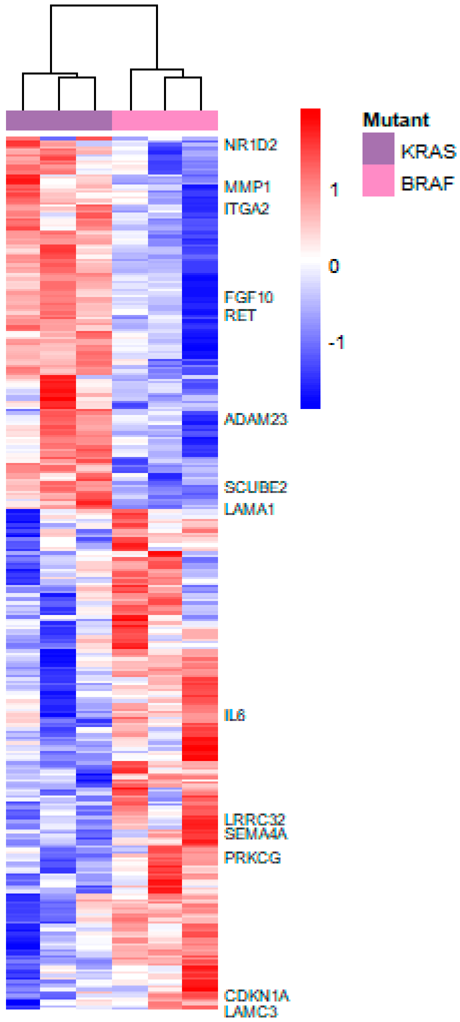

Figure S9. Differential gene expression in AAH samples with *BRAF* or *KRAS* mutations. mRNA profiles of *BRAF*- and *KRAS*-mutant AAH samples clustered by complete linkage and correlation similarity. The gene names are obtained from Sivakumar *et al.* (2017) and cancer-related pathway MSigDB cancer gene sets (http://software.broadinstitute.org/gsea/msigdb/index.jsp). Samples are presented in columns and mRNA expression in rows. AAH, atypical adenomatous hyperplasia; ADC, adenocarcinoma; NOR, normal.
