# Supplementary material for: Gene Expression Profiles of Multiple Synchronous Lesions in Lung Adenocarcinoma
Source: Cells. 2021 Dec 10;10(12):3484. doi: 10.3390/cells10123484 (PMC8700398; doi:10.3390/cells10123484)
Supplement: Supplementary file 1 [file cells-10-03484-s001.zip › cells-supplementary-tables-20211204.pdf]

Table S1. Patient characteristics and specimen information

Patient[1]	Age	Sex	Smoking (pack-year)	Lesion	(P = 9, n = 38)
P1	64	M	CS (20)	NOR	NOR
				AAH	AAH2, AAH3, AAH4
				ADC	ADC2
P2	75	M	ES (35)	NOR	NOR
				AAH	AAH
				ADC	ADC2
P3	66	M	ES (10)	NOR	NOR
				AAH	AAH1, AAH2
				ADC	ADC
P4	55	F	NS (0)	NOR	NOR
				AAH	AAH
				ADC	ADC
P5	53	F	NS (0)	NOR	NOR
				AAH	AAH1, AAH2
				ADC	ADC
P8	69	M	ES (30)	NOR	NOR
				AAH	AAH1, AAH2, AAH3, AAH4, AAH6
				ADC	ADC
P9	70	F	NS (0)	NOR	NOR
				AAH	AAH
				ADC	ADC
P10	72	M	CS (50)	NOR	NOR
				AAH	AAH
				ADC	ADC
P11	51	M	ES (12)	NOR	NOR
				AAH	AAH1, AAH2
				ADC	ADC

CS: current smoker, ES: ex-smoker, NS: never smoker; AAH: atypical adenomatous hyperplasia; ADC: adenocarcinoma; NOR: non-tumorous tissues

[1]Patient specimens were collected as part of a previous targeted sequencing and whole exome study and the same patient identification number was used.

Table S2. Primer sequences

Gene	RefSeq records	Sequences
ACSL5	NM_016234.3, NM_203379.1, NM_203380.1	(F): 5'-CTCAACCCGTCTTACCTCTTC-3'<br>(R): 5'-CCTCATACATAGTCTTGGCATC-3'
CCBE1	NM_133459.4	(F): 5'-TACCGATATGACCGGGAGAG-3'<br>(R): 5'-AGCTGCCCAAGGTATTGATG-3'
SERINC2	NM_001199039.1, NM_001199038.1, NM_001199037.1, NM_018565.3, NM_178865.4	(F): 5'-ACCCTCTACACCATGTTTGTC-3'<br>(R): 5'-CACTGGGTCTCATAGCCCTC-3'
GAPDH	NM_002046.7, NM_001289745.3	(F): 5'-GAAGGTGAAGGTCGGAGTC-3'<br>(R): 5'-GAAGATGGTGATGGGATTTC-3'

Table S3. Histochemical scoring assessment (H-score)

Patient	Label	Lesions	ACSL5	FUT2	SERINC2	DLX3	CCBE1
P1	NOR*	normal	130**		100		
	AAH2*	AAH	154**		195		
	AAH4*	AAH	210		200		
	ADC1	ADC-lepidic	245**		202		
	ADC2*	ADC-non lepidic	265**		205		
P2	NOR*	normal		5		5**	
	AAH*	AAH		50		20**	
	AIS	AIS		80		40	
	ADC1	ADC-lepidic		90		100**	
	AAH2*	ADC-non lepidic		100		110**	
P4	NOR*	normal		0		0	
	AAH*	AAH		190**		210**	
	ADC*	ADC-non lepidic		170**		175**	
P5	NOR*	normal		0			0
	AAH1*	AAH		95**			0
	AAH2*	AAH		30**			0
	ADC*	ADC		195**			0
P8	NOR*	normal	120		130**		
	AAH3*	AAH	210		200**		
	ADC*	ADC	225		205**		
P9	NOR*	normal	70		15		
	AAH*	AAH	192		192		
	ADC*	ADC	220		192		
P10	NOR*	normal	30**		60		
	ADC*	ADC	280**		130		
P11	NOR*	normal	170**		10		
	AAH1*	AAH	210**		80**		
	AAH2*	AAH	220**		70**		
	ADC*	ADC	230**		130**		
P18	NOR	normal		0**			0**
	AAH1	AAH		10**			0
	ADC	ADC		220**			0**

*Samples used in RNA-seq analysis

**Samples used in IHC analysis

Table S4. Differential expression of genes which were overlapped with Sivakumar et al, 2017 study results (n=269)

Gene	Fold change (log2)			
	AAH vs NOR	ADC vs NOR	ADC vs AAH	Cluster[1]
SLIT2	-0.75	-1.65	-0.90	1
DACH1	-0.86	-2.43	-1.57	1
CCBE1	-0.92	-2.14	-1.22	1
COL13A1	-0.97	-1.81	-0.84	1
GRIA1	-1.03	-1.97	-0.94	1
BCL6B	-1.05	-2.73	-1.69	1
FIBIN	-1.09	-2.39	-1.30	1
NPR3	-0.54	-1.25	-0.71	1
RPL23AP32	-0.47	-1.24	-0.76	1
ITIH3	-0.44	-1.21	-0.78	1
MFAP4	-0.63	-1.52	-0.89	1
CRYAB	-0.48	-1.42	-0.94	1
PKDCC	-0.74	-1.69	-0.96	1
IL18R1	-0.41	-1.38	-0.96	1
NKD1	-0.75	-1.73	-0.98	1
LEPR	-0.47	-1.44	-0.98	1
OLFML2A	-0.96	-1.95	-0.99	1
VEGFC	-0.59	-1.59	-1.00	1
LTBP4	-0.53	-1.56	-1.03	1
THBD	-0.38	-1.42	-1.03	1
GDF10	-0.59	-1.65	-1.06	1
HEG1	-0.61	-1.68	-1.06	1
ERG	-0.56	-1.64	-1.08	1
SASH1	-0.42	-1.52	-1.09	1
RAPGEF4	-0.52	-1.63	-1.11	1
JAM2	-0.42	-1.57	-1.14	1
PKNOX2	-0.68	-1.83	-1.15	1
PALMD	-0.67	-1.83	-1.16	1
ADAMTS8	-0.95	-2.12	-1.17	1
HIGD1B	-0.67	-1.88	-1.21	1
BMPER	-0.82	-2.06	-1.24	1
FGFR4	-0.73	-1.99	-1.25	1
ADRA1A	-0.79	-2.06	-1.26	1
GRASP	-0.64	-1.90	-1.26	1
IGSF10	-0.71	-1.99	-1.28	1
KCTD16	-0.70	-1.98	-1.28	1
COX4I2	-0.50	-1.79	-1.28	1
STARD9	-0.55	-1.83	-1.28	1
ARHGEF4	-0.66	-1.95	-1.29	1
S1PR1	-0.63	-1.91	-1.29	1
CDO1	-0.79	-2.08	-1.29	1
COL5A3	-0.80	-2.11	-1.31	1

IL12A	-0.77	-2.10	-1.33	1
PDGFB	-0.57	-1.93	-1.37	1
GBP4	-0.71	-2.08	-1.37	1
CLEC1A	-0.72	-2.10	-1.38	1
LDB2	-0.59	-1.99	-1.40	1
AFAP1L1	-0.68	-2.12	-1.44	1
THSD1	-0.63	-2.08	-1.44	1
NKG7	-0.70	-2.15	-1.44	1
EGFL7	-0.65	-2.10	-1.45	1
GRK5	-0.58	-2.03	-1.45	1
FLT4	-0.64	-2.11	-1.46	1
CLEC14A	-0.79	-2.26	-1.47	1
TOX2	-0.63	-2.12	-1.49	1
CD93	-0.74	-2.24	-1.50	1
KIAA1462	-0.58	-2.08	-1.50	1
NCKAP5	-0.86	-2.36	-1.51	1
SEMA5A	-0.64	-2.16	-1.52	1
SCUBE1	-0.69	-2.22	-1.53	1
ADARB2	-0.96	-2.51	-1.55	1
SGCG	-0.92	-2.50	-1.59	1
NRN1	-0.58	-2.17	-1.59	1
GIMAP5	-0.75	-2.34	-1.60	1
RAMP2	-0.67	-2.27	-1.60	1
CSPG4	-0.60	-2.25	-1.64	1
KCNMB4	-0.64	-2.32	-1.68	1
ANO2	-0.77	-2.45	-1.68	1
NOVA2	-0.80	-2.49	-1.69	1
MYCT1	-0.72	-2.42	-1.70	1
CLEC3B	-0.75	-2.45	-1.70	1
RASIP1	-0.73	-2.48	-1.75	1
ARHGEF15	-0.67	-2.48	-1.81	1
GPIHBP1	-1.39	-3.26	-1.87	1
PKHD1L1	-0.76	-2.66	-1.91	1
KCNK3	-0.81	-2.77	-1.96	1
RXFP1	-0.75	-2.74	-1.99	1
SERTM1	-1.13	-3.21	-2.09	1
CLDN5	-1.13	-3.62	-2.49	1
TMIE	-0.85	-1.23	-0.37	1
ST6GALNAC5	-0.40	-1.26	-0.85	1
GRAP	-0.40	-1.40	-1.01	1
LINC00312	-0.45	-1.60	-1.15	1
RGS6	-1.04	-1.89	-0.85	1
DPP6	-1.39	-2.53	-1.14	1
APLN	-0.76	-2.70	-1.93	1
ATOH8	-0.27	-1.41	-1.14	2
LAMP3	0.48	-0.80	-1.29	2
GKN2	0.34	-1.05	-1.39	2

FHL5	-0.20	-1.61	-1.40	2
SFTPC	0.53	-0.93	-1.45	2
SFTPA1	0.61	-0.86	-1.46	2
ACADL	0.04	-1.56	-1.60	2
SLC51B	0.17	-1.45	-1.62	2
WISP2	-0.21	-1.87	-1.66	2
CLDN18	0.19	-1.62	-1.81	2
SFTPA2	0.61	-1.36	-1.98	2
MGAT3	-0.46	-2.48	-2.02	2
LGI3	0.20	-1.83	-2.03	2
UPK3B	-0.54	-2.58	-2.04	2
CRTAC1	0.78	-1.41	-2.19	2
PRKG2	0.14	-2.13	-2.27	2
RS1	-0.36	-3.10	-2.74	2
SLC16A12	0.78	-2.15	-2.93	2
LOC283299	-0.26	-1.38	-1.12	2
CA10	3.77	6.56	2.79	3
GCNT3	3.65	6.01	2.36	3
COL10A1	3.26	5.73	2.47	3
ATP10B	2.82	4.67	1.85	3
B3GNT3	2.46	3.36	0.90	3
TMPRSS4	2.42	4.57	2.15	3
FUT2	2.40	4.14	1.74	3
TOX3	2.17	3.54	1.36	3
METTL7B	2.14	3.31	1.17	3
PKHD1	2.10	3.06	0.96	3
EPHA10	1.99	4.26	2.27	3
C1orf53	1.75	2.47	0.73	3
ABCC3	1.74	3.04	1.30	3
KCNQ3	1.57	2.34	0.76	3
ST6GALNAC1	1.51	2.69	1.18	3
LAD1	1.47	2.02	0.56	3
ETV4	1.41	3.48	2.07	3
KIF26B	1.38	2.75	1.37	3
SPDEF	1.33	3.17	1.84	3
SERINC2	1.26	2.32	1.06	3
TMEM184A	1.19	1.85	0.66	3
NT5E	1.09	2.48	1.39	3
SPTBN2	1.00	2.53	1.53	3
KCNK1	0.97	1.42	0.45	3
TMEM45B	0.89	1.54	0.65	3
TMED3	0.89	1.31	0.42	3
AP1M2	0.87	1.29	0.42	3
TXNDC17	0.83	1.45	0.62	3
PYCR1	0.80	2.42	1.62	3
GOLM1	0.80	1.91	1.11	3
RAB25	0.78	1.61	0.83	3

EPCAM	0.73	1.91	1.17	3
PRSS16	0.71	1.56	0.85	3
EHF	0.71	1.55	0.85	3
OCIAD2	0.69	1.58	0.89	3
ZDHHC13	0.65	1.26	0.60	3
ENC1	0.62	1.56	0.94	3
ACSL5	0.62	1.90	1.28	3
COL17A1	1.36	4.11	2.75	3
ABCA12	2.51	4.75	2.23	3
RASAL1	0.78	2.82	2.04	3
CEACAM6	0.81	2.66	1.84	3
CLIC6	1.27	3.04	1.76	3
PLD5	1.40	3.03	1.63	3
ARHGEF19	0.57	2.07	1.50	3
KIF20A	0.72	2.20	1.48	3
PSAT1	0.78	2.25	1.47	3
PLEKHN1	0.73	2.18	1.45	3
LRAT	1.44	2.89	1.45	3
LRIG3	0.54	1.88	1.35	3
MACC1	0.62	1.84	1.22	3
LAPTM4B	0.77	1.95	1.19	3
C1orf106	0.50	1.68	1.18	3
TK1	0.84	2.00	1.16	3
SNORA10	0.59	1.72	1.14	3
KCNK5	0.72	1.85	1.13	3
RHPN1	0.43	1.48	1.05	3
FERMT1	0.61	1.64	1.03	3
F2RL1	0.70	1.69	1.00	3
UGT8	0.96	1.94	0.98	3
MAL2	0.77	1.73	0.96	3
ARHGEF16	0.50	1.44	0.94	3
TFAP2C	0.63	1.52	0.89	3
GRHL2	0.45	1.25	0.80	3
SLC25A10	0.78	1.55	0.77	3
SRD5A3	0.49	1.23	0.74	3
ATP1B1	0.67	1.34	0.67	3
ENO1	0.60	1.24	0.64	3
TLCD1	0.63	1.23	0.60	3
CXCL13	2.96	4.67	1.71	3
BAAT	2.22	3.27	1.05	3
CST2	1.37	2.96	1.59	3
ANKRD22	1.27	2.72	1.45	3
EPN3	1.41	2.60	1.19	3
BCL2L15	0.97	1.94	0.97	3
COCH	1.06	1.79	0.73	3
GOLGA7B	0.79	1.78	0.99	3
AGR2	1.02	1.76	0.74	3

PAFAH1B3	0.65	1.67	1.03	3
ADORA2B	0.68	1.47	0.78	3
KDELR3	0.76	1.45	0.70	3
SNORA74A	0.61	1.40	0.79	3
SNORA71A	0.84	1.31	0.48	3
REEP6	0.43	1.28	0.85	3
VWA2	0.62	1.27	0.65	3
CD180	0.80	1.25	0.45	3
UNC5CL	0.73	1.24	0.51	3
ZDHHC9	0.65	1.21	0.55	3
NRIP3	0.53	5.62	5.09	4
NPTX1	-0.35	4.61	4.96	4
COL22A1	0.09	3.69	3.60	4
CRABP2	-0.22	2.86	3.08	4
MEX3A	-1.26	1.73	3.00	4
SRCIN1	-0.40	1.83	2.23	4
KIF4A	-0.20	2.02	2.22	4
SCUBE3	0.72	2.87	2.15	4
NUP210L	0.63	2.78	2.15	4
CDC20	-0.08	2.01	2.08	4
HIST1H2AJ	-0.39	1.67	2.06	4
TUBB3	-0.58	1.49	2.06	4
TTK	0.21	2.27	2.06	4
KRT80	0.02	2.06	2.04	4
SIX4	0.15	2.19	2.04	4
EEF1A2	0.55	2.54	1.99	4
CLDN1	0.30	2.28	1.98	4
SNORA69	0.07	2.03	1.96	4
MKI67	0.01	1.90	1.89	4
ASPM	-0.20	1.68	1.88	4
GREB1	-0.06	1.80	1.85	4
FAM111B	-0.44	1.38	1.82	4
COL1A1	-0.04	1.78	1.82	4
CHRNA5	-0.33	1.45	1.78	4
HOXD4	0.44	2.13	1.69	4
COL3A1	0.04	1.70	1.65	4
LGR4	0.29	1.94	1.65	4
DLGAP5	0.32	1.96	1.63	4
RRM2	0.03	1.57	1.54	4
TFAP2A	-0.23	1.30	1.53	4
DIO2	-0.01	1.48	1.49	4
BUB1B	0.03	1.51	1.48	4
HIST1H3B	0.00	1.47	1.47	4
CCNB2	-0.50	0.95	1.46	4
NQO1	0.26	1.71	1.46	4
LGI2	-0.07	1.38	1.45	4
MDK	0.06	1.49	1.42	4

THBS2	-0.01	1.38	1.39	4
MCM10	-0.43	0.95	1.38	4
FHL2	-0.40	0.92	1.32	4
STIL	0.11	1.42	1.31	4
MFAP2	0.10	1.35	1.24	4
FAP	0.31	1.20	0.89	4
LHFPL4	1.05	4.09	3.03	4
HIST1H4L	0.55	2.12	1.57	4
DNAJC12	0.37	1.84	1.47	4
IL6	-2.86	-3.67	-0.81	5
CCDC81	-0.98	-1.23	-0.25	5
CCDC17	-1.12	-1.52	-0.40	5
DLEC1	-1.36	-1.61	-0.25	5
DCDC1	-1.47	-1.95	-0.48	5
HABP2	6.01	6.54	0.53	7
CEACAM8	4.19	3.61	-0.58	7
GDF15	3.31	3.61	0.30	7
CRYM	3.12	4.07	0.95	7
CRLF1	2.84	2.94	0.10	7
MMP7	2.76	2.89	0.13	7
SCTR	1.98	2.32	0.34	7
GLS2	1.94	2.20	0.26	7
ACSM3	1.58	1.98	0.40	7
WNT4	1.57	2.10	0.53	7
RAP1GAP	1.52	1.54	0.02	7
CLEC5A	1.39	1.80	0.41	7
GGT5	1.39	1.55	0.16	7
CXCL17	1.37	1.54	0.17	7
HPN	1.37	1.14	-0.22	7
MUC1	1.35	1.74	0.39	7
LMO3	1.30	1.41	0.11	7
TMEM92	1.28	1.70	0.42	7
CDH3	1.24	1.37	0.13	7
GOLT1A	1.20	1.36	0.17	7
MGST1	1.10	1.22	0.12	7
FOXA3	2.36	3.10	0.73	7
CTSE	1.37	1.72	0.36	7
PLA2G10	1.11	1.28	0.17	7
AGBL1	1.00	-0.88	-1.88	8

[1]Cluster number denotes the eight distinct expression pattern across normal to AAH, and to ADC; cluster 1, ↘↘; Cluster 2, →↘; Cluster 3, ↗↗; Cluster 4, →↗; Cluster 5, ↘→; Cluster 6, ↘↗; Cluster 7, ↗→; Cluster8, ↗↘

AAH, atypical adenomatous hyperplasia; ADC, adenocarcinoma; NOR, normal lung sample;

Table S5. Differentially expressed genes which were overlapped with the PanCancer Immune Profiling Panel gene lists (n=145)

Gene	Fold change (log2)			Cluster[1]
	AAH vs NOR	ADC vs NOR	ADC vs AAH	
IL1B	-1.15	-1.85	-0.70	1
ABCB1	-1.17	-1.84	-0.67	1
TNFAIP3	-1.26	-2.04	-0.77	1
IRF1	-1.47	-2.12	-0.65	1
NFKBIA	-0.65	-1.43	-0.78	1
ETS1	-0.35	-1.25	-0.90	1
NLRP3	-0.61	-1.53	-0.92	1
IL18R1	-0.41	-1.38	-0.96	1
FEZ1	-0.42	-1.39	-0.97	1
MAPK11	-0.61	-1.59	-0.98	1
VEGFC	-0.59	-1.59	-1.00	1
THBD	-0.38	-1.42	-1.03	1
MCAM	-0.43	-1.50	-1.06	1
LILRB2	-0.68	-1.75	-1.07	1
LILRA5	-0.60	-1.70	-1.10	1
ITGA5	-0.50	-1.61	-1.11	1
LILRA1	-0.63	-1.76	-1.13	1
IL1R2	-0.85	-2.00	-1.16	1
ITGA2B	-0.45	-1.67	-1.22	1
LRRN3	-0.47	-1.73	-1.26	1
IL12A	-0.77	-2.10	-1.33	1
CX3CL1	-0.79	-2.15	-1.36	1
MEFV	-0.77	-2.19	-1.42	1
MME	-0.66	-2.26	-1.60	1
CXCR2	-0.52	-1.24	-0.72	1
PDCD1LG2	-0.43	-1.24	-0.81	1
KLRK1	-0.37	-1.24	-0.87	1
HSD11B1	-0.71	-1.25	-0.54	1
CREB5	-0.78	-1.36	-0.58	1
CD34	-0.75	-1.37	-0.63	1
IL5RA	-0.68	-1.46	-0.78	1
IL1RL1	-0.76	-1.54	-0.78	1
FPR2	-0.93	-1.57	-0.64	1
TNFRSF4	-1.03	-1.61	-0.58	1
CDKN1A	-0.87	-1.64	-0.76	1
IL1A	-0.81	-1.70	-0.89	1
S100A8	-0.80	-1.77	-0.97	1
KIR3DL2	-0.60	-1.78	-1.18	1
XCL2	-0.85	-1.88	-1.03	1
CCL16	-0.58	-1.94	-1.35	1
CCL14	-0.82	-2.16	-1.34	1
IL3RA	-0.98	-2.17	-1.18	1

CLEC6A	-0.68	-2.24	-1.57	1
CXCR1	-1.09	-2.26	-1.17	1
ARG1	-1.91	-3.06	-1.15	1
CSF3	-1.88	-3.07	-1.19	1
CCL26	-1.31	-3.08	-1.78	1
NFATC1	-0.33	-1.30	-0.97	2
ICAM2	-0.35	-1.36	-1.02	2
PRF1	-0.37	-1.41	-1.05	2
KLRD1	-0.35	-1.47	-1.11	2
CX3CR1	-0.30	-1.42	-1.12	2
MARCO	-0.18	-1.39	-1.21	2
CFP	-0.28	-1.50	-1.22	2
CFD	-0.16	-1.39	-1.23	2
TREM1	0.10	-1.16	-1.25	2
LAMP3	0.48	-0.80	-1.29	2
SLC11A1	0.14	-1.21	-1.34	2
IL18RAP	-0.39	-1.78	-1.38	2
PECAM1	-0.43	-1.82	-1.39	2
CXCL2	-0.06	-1.45	-1.39	2
MASP1	-0.31	-1.81	-1.50	2
GZMH	-0.54	-2.05	-1.51	2
KLRF1	-0.38	-1.89	-1.52	2
GZMB	-0.55	-2.09	-1.54	2
TAL1	-0.01	-1.60	-1.58	2
TBX21	-0.38	-1.98	-1.60	2
CTSW	-0.13	-1.82	-1.69	2
CSF3R	0.72	-1.06	-1.78	2
CAMP	-0.32	-2.22	-1.90	2
CDH5	-0.47	-2.37	-1.90	2
KIR2DL3	-0.59	-2.68	-2.09	2
GNLY	-0.54	-2.63	-2.10	2
CD160	-0.46	-2.61	-2.15	2
CD36	-0.48	-2.65	-2.17	2
NCR1	-0.37	-2.59	-2.22	2
KIR2DL1	-0.27	-3.22	-2.95	2
KIR3DL1	-0.62	-3.89	-3.27	2
SH2D1B	-0.20	-3.75	-3.55	2
EGR1	-0.30	-1.40	-1.10	2
CCL3L1	-0.04	-2.55	-2.51	2
CD24	2.39	3.45	1.06	3
DPP4	1.87	2.75	0.88	3
NT5E	1.09	2.48	1.39	3
EPCAM	0.73	1.91	1.17	3
MNX1	2.91	5.61	2.70	3
CD1A	1.56	3.65	2.09	3
CEACAM6	0.81	2.66	1.84	3
CEACAM1	0.81	2.20	1.39	3

S100B	1.21	2.53	1.32	3
TNFSF15	1.00	2.23	1.24	3
F2RL1	0.70	1.69	1.00	3
IL22RA2	2.27	4.73	2.46	3
CXCL13	2.96	4.67	1.71	3
CR2	1.56	3.39	1.83	3
CD19	0.98	2.72	1.73	3
PAX5	0.89	2.45	1.56	3
PMCH	1.03	2.34	1.31	3
CCL17	1.41	2.18	0.77	3
C5	1.30	2.04	0.74	3
FOXP3	1.13	1.98	0.85	3
CCL22	1.32	1.91	0.59	3
MS4A1	0.68	1.91	1.22	3
SPINK5	1.02	1.91	0.89	3
BLK	0.72	1.72	1.00	3
DMBT1	0.53	1.40	0.87	3
CCR6	0.71	1.38	0.67	3
HLA-DOB	0.54	1.38	0.84	3
IL2RA	0.57	1.26	0.69	3
CD180	0.80	1.25	0.45	3
CSF2	0.57	1.21	0.65	3
CXCL14	0.86	4.44	3.58	4
SPP1	0.94	4.40	3.46	4
C8A	0.10	2.47	2.37	4
TTK	0.21	2.27	2.06	4
COL3A1	0.04	1.70	1.65	4
IL1RL2	-0.29	1.30	1.58	4
CCL28	0.09	1.47	1.38	4
CDH1	0.43	1.65	1.21	4
CDK1	0.28	1.25	0.97	4
LTB	0.22	1.22	1.00	4
CCL2	-1.36	-1.46	-0.10	5
CCL21	-1.44	-1.68	-0.24	5
CXCL10	-1.57	-1.44	0.13	5
CXCL9	-2.25	-1.57	0.68	5
IFNG	-2.36	-2.82	-0.46	5
IL6	-2.86	-3.67	-0.81	5
C9	-3.01	-1.53	1.48	5
TNF	-1.04	-1.27	-0.23	5
SELE	-1.59	-1.98	-0.39	5
CXCL8	-1.72	-2.18	-0.46	5
CXCL11	-1.85	-2.22	-0.37	5
ADORA2A	-2.35	-2.78	-0.43	5
FUT5	-2.83	-3.38	-0.55	5
CCL7	-4.08	-0.20	3.88	6
CEACAM8	4.19	3.61	-0.58	7

C4BPA	2.34	2.00	-0.33	7
IL13RA2	2.08	2.41	0.33	7
CLEC5A	1.39	1.80	0.41	7
MUC1	1.35	1.74	0.39	7
CTSH	1.23	0.89	-0.34	7
TLR10	1.33	1.80	0.47	7
CD79A	1.33	1.78	0.45	7
TNFRSF17	1.79	1.61	-0.18	7
LTK	0.89	-0.68	-1.57	8

[1]Cluster number denotes the eight distinct expression pattern across normal to AAH, and to ADC; cluster 1, ↘↘; Cluster 2, →↘; Cluster 3, ↗↗; Cluster 4, →↗; Cluster 5, ↘→; Cluster 6, ↘↗; Cluster 7, ↗→; Cluster8, ↗↘

AAH, atypical adenomatous hyperplasia; ADC, adenocarcinoma; NOR, normal lung sample.

Table S6. Annotated biological process of up-regulated DEGs which were overlapped with the PanCancer Panel (n=48)

**Categories[1]**	**Gene**	**Cluster (%)[2]**	**Log2 Fold change of AAH / NOR**	**Log2 Fold change of ADC / NOR**
Adhesion	*CEACAM1, CEACAM6, EPCAM, CEACAM8*	3 (75), 7 (25)	1.64	2.6
Antigen Processing	*HLA-DOB*	3 (100)	0.54	1.38
B-Cell Functions	*BLK, CD19, CR2, MS4A1*	3 (100)	0.98	2.44
Cell Functions	*CD1A, EPCAM, PMCH, TNFRSF17*	3 (75), 7 (25)	1.28	2.38
Chemokines	*CCL17, CCL22, CXCL13, IL22RA2, TNFSF15, CEACAM8, IL13RA2, CCL28, CXCL14*	3 (56), 4 (22), 7 (22)	1.8	3.07
Complement	*C5, C4BPA, C8A*	3 (33), 4 (33), 7 (33)	1.25	2.17
Cytokines	*FOXP3, HLA-DOB, LTB, SPP1*	3 (50), 4 (50)	0.71	2.25
Macrophage Functions	*CSF2*	3 (100)	0.57	1.21
Pathogen Defense	*CCL22*	3 (100)	1.32	1.91
Regulation	*CD19, CSF2, DPP4, IL2RA, SPINK5, CDH1, COL3A1*	3 (71), 4 (29)	0.78	1.89
T-Cell Functions	*DPP4, F2RL1, FOXP3, IL13RA2*	3 (75), 7 (25)	1.45	2.21
TLR	*TLR10*	7 (100)	1.33	1.8
TNF Superfamily	*TNFSF15, TNFRSF17, LTB*	3 (33), 4 (33), 7 (33)	1	1.69
Transporter Functions	*NT5E*	3 (100)	1.09	2.48

[1]Categories are from the PanCancer Immune Profiling Panel Gene Ontology information.

[2]Cluster is assigned to cluster name according to the previous DEG analysis and its percentage represents the proportion of the number of genes in each cluster to the number of all genes in each category.

AAH, atypical adenomatous hyperplasia; ADC, adenocarcinoma; NOR, normal lung sample

Table S7. Annotated biological process of down-regulated DEGs which were overlapped with the PanCancer Panel (n=91)

**Categories**[1]	**Gene**	**Cluster (%)**[2]	**Log2 fold change of AAH / NOR**	**Log2 fold change of ADC / NOR**
Adhesion	*ICAM2, ITGA2B, ITGA5, MCAM*	1 (75), 2 (25)	-0.43	-1.54
Cell Cycle	*CDKN1A*	1 (100)	-0.87	-1.64
Cell Functions	*ADORA2A, CSF3, FEZ1, GZMB, GZMH, HSD11B1, IL3RA, KLRF1, MME, NCR1*	1 (50), 2 (40), 5 (10)	-0.88	-2.15
Chemokines	*CCL2, CCL7, CCL14, CCL16, CCL21, CCL26, CX3CL1, CXCL2, CXCL8, CXCL10, CXCL11, CXCR1, CXCR2, CX3CR1, IL1B, IRF1, XCL2*	1 (53), 2 (12), 5 (29), 6 (6)	-1.23	-1.81
Complement	*C9*	5 (100)	-3.01	-1.53
Cytokines	*CCL3L1, CXCL8, CXCL9, CXCL10, IL1A, IL12A, IL1B, IL1R2, IL5RA*	1 (56), 2 (11), 5 (33)	-1.09	-1.87
Cytotoxicity	*GNLY, GZMB, GZMH, PRF1*	2 (100)	-0.5	-2.04
Interleukins	*CXCL8, IL6, IL1A, IL12A, IL1B, TNF*	1 (50), 5 (50)	-1.39	-2.13
Leukocyte Functions	*SH2D1B, THBD*	1 (50), 2 (50)	-0.29	-2.58
Microglial Functions	*CX3CR1*	2 (100)	-0.3	-1.42
NK Cell Functions	*IL12A, KIR2DL1, KIR2DL3, KIR3DL1, KIR3DL2, KLRD1, KLRF1, KLRK1, NCR1*	1 (33), 2 (67)	-0.48	-2.32
Pathogen Defense	*CXCL8, CXCL10, IL1B, GNLY, PRF1*	1 (20), 2 (40), 5 (40)	-1.07	-1.9
Regulation	*CCL16, CD34, CDKN1A, CXCR1, CXCR2, IL12A, IL1B, IRF1, KIR3DL2, KLRK1, LILRA1, LILRB2, CCL21,CXCL10, CXCL8, CXCL9, SELE, CD160, CXCL2, KIR2DL1, KIR2DL3, KIR3DL1, ICAM2, KLRD1, NFATC1, TAL1, TBX21*	1 (44), 2 (37), 5 (19)	-0.8	-1.91
Senescence	*CDKN1A, EGR1, ETS1, IRF1*	1 (75), 2 (25)	-0.75	-1.6
T-Cell Functions	*CX3CL1, CXCL9, CXCL10, CXCL11, EGR1, IL12A, IL18R1, IL18RAP, TBX21*	1 (33), 2 (33), 5 (33)	-0.97	-1.78
TNF Superfamily	*TNF, TNFAIP3, TNFRSF4*	1 (67), 5 (33)	-1.11	-1.64
Transporter Functions	*CD36, CTSW, PECAM1*	2 (100)	-0.35	-2.1

[1]Categories are from the PanCancer Immune Profiling Panel Gene Ontology information.

[2]Cluster is assigned to cluster name according to the previous DEG analysis and its percentage represents the proportion of the number of genes in each cluster to the number of all genes in each category.

AAH, atypical adenomatous hyperplasia; ADC, adenocarcinoma; NOR, normal lung sample.
